# Lifestyle and psychosocial factors in inflammatory bowel disease: Prevalence, impact, motivation, and support needs

**DOI:** 10.1371/journal.pone.0331092

**Published:** 2025-08-29

**Authors:** Karlijn Demers, Evelien M. B. Hendrix, Ashkan Rezazadeh Ardabili, Quirine M. Bredero, Ad A. van Bodegraven, Daniëlle van der Horst, Daisy M. A. E. Jonkers, Merel L. Kimman, Zlatan Mujagic, Mariëlle J. Romberg-Camps, Tessa E. H. Römkens, Menne P. Scherpenzeel, Maya J. Schroevers, Laurents P. S. Stassen, Rachel L. West, Gerard Dijkstra, Marieke J. Pierik

**Affiliations:** 1 Department of Gastroenterology and Hepatology, Maastricht University Medical Centre+, Maastricht, The Netherlands; 2 Department of Surgery, Maastricht University Medical Centre+, Maastricht, The Netherlands; 3 Research Institution for Nutrition and Translational Research in Metabolism (NUTRIM), Maastricht University, Maastricht, The Netherlands; 4 Department of Health Psychology, University Medical Centre Groningen, University of Groningen, Groningen, The Netherlands; 5 Department of Gastroenterology-Geriatrics-Internal and Intensive Care Medicine Co-MIK, Zuyderland Medical Centre, Heerlen-Sittard-Geleen, The Netherlands; 6 Crohn & Colitis NL, Woerden, The Netherlands; 7 Clinical Epidemiology and Medical Technology Assessment, Maastricht University Medical Centre+, Maastricht, The Netherlands; 8 Care and Public Health Research Institute (CAPHRI), Maastricht University, Maastricht, The Netherlands; 9 Department of Gastroenterology and Hepatology, Jeroen Bosch Hospital, ‘s-Hertogenbosch, The Netherlands; 10 Department of Gastroenterology and Hepatology, Franciscus Gasthuis en Vlietland, Rotterdam, The Netherlands; 11 Department of Gastroenterology and Hepatology, University Medical Centre Groningen, Groningen, The Netherlands; Zanjan University of Medical Sciences, IRAN, ISLAMIC REPUBLIC OF

## Abstract

**Background and aim:**

Lifestyle and psychosocial factors impact mucosal inflammation and well-being of Inflammatory Bowel Disease (IBD) patients. However, lifestyle assessment and interventions are not standard care. The aim of this study was to estimate the occurrence of and gather patients’ perspectives on unfavorable lifestyle and psychosocial factors in individuals with IBD.

**Methods:**

A multicenter study was conducted, enrolling IBD patients using a telemedicine platform that reports on disease activity, lifestyle, and psychosocial factors. Patients' perspectives were gathered through a nationwide online survey distributed by the Dutch IBD patient organization.

**Results:**

In the telemedicine cohort (*n* = 460), 16.3% followed a specific diet, and 50.7% believed diet impacted their disease or quality of life. Additionally, 67.4% did not meet exercise norms, 9.3% smoked, and 8.0% had excessive alcohol consumption (>7 units/week). About one-third experienced high stress, poor sleep regularly, and emotional distress occasionally. In the nationwide survey (*n* = 1126), most patients (58–91%) believed that stress, unhealthy diet, poor sleep, physical inactivity, and anxiety or depression could cause intestinal symptoms. Around 70% were motivated to change diet, stress management, and physical activity. Less than one-fifth of patients received hospital support, with the majority being satisfied. Approximately 20% of patients desired but lacked support concerning stress, physical activity, diet, and sleep.

**Conclusions:**

Patients with IBD commonly report unfavorable lifestyle and psychosocial factors, recognize their impact on intestinal symptoms, and are motivated to change, but often lack hospital support. This underscores the importance for systematic incorporation of lifestyle and psychosocial factors into patient-centered IBD care and the potential for targeted interventions.

## Introduction

Inflammatory Bowel Disease (IBD) is characterized by chronic inflammation of the intestinal tract, affecting millions of individuals worldwide. The chronic nature and fluctuating course of the disease, with debilitating symptoms occurring during flare-ups and often even in remission, pose substantial challenges for both patients and healthcare systems [1]. IBD-related symptoms, including abdominal pain, diarrhea, fatigue, or urgency, often disrupt a person’s daily routine. These disruptions can impact various aspects of life, such as dietary choices, exercise regimen, sleep patterns, and emotional well-being [2–5]. In addition, these lifestyle and psychosocial factors have been recognized as important determinants of flares, chronic abdominal pain, and quality of life in patients with IBD [6–8]. This creates a bidirectional relationship in which IBD symptoms can negatively influence lifestyle behaviors and psychosocial well-being. In turn, unfavorable lifestyle and psychosocial factors, such as poor diet, physical inactivity, excessive alcohol consumption, smoking, high levels of stress, poor sleep, emotional distress, and lack of social support, may increase disease severity and burden [9–12]. This dynamic perpetuates a challenging cycle. However, current international guidelines often lack detailed recommendations for systematically incorporating lifestyle and psychosocial factors into clinical practice [13–16], which poses a challenge for developing comprehensive IBD care. Consequently, long-standing unfavorable lifestyle or psychosocial factors may contribute to negative overall health effects and adversely affect the clinical course of IBD.

Gaining a deeper understanding of the occurrence of lifestyle and psychosocial factors, along with patients’ perspectives on these factors, is crucial for optimizing interventional strategies [17]. This insight will help improve comprehensive and personalized disease management that incorporates lifestyle modifications and psychosocial support [18]. By understanding which factors are most common, impactful, and prioritized by patients, healthcare systems could develop more patient-centered and targeted support services and allocate resources more effectively [18]. Assessing patients’ perspectives is also essential in tailoring interventions that resonate with their needs. Such tailored interventions should assist patients in achieving healthy lifestyle changes, ultimately leading to improved disease outcomes and quality of life [19,20].

In this study, we aimed to estimate the occurrence of unfavorable lifestyle and psychosocial factors in a multicenter outpatient cohort of patients with IBD. Additionally, through a collaborative survey with the Dutch IBD patient organization Crohn & Colitis Netherlands (NL), we aimed to gather patients’ perspectives from a cohort of patients with IBD on the impact of these factors, motivation, and support for change.

## Materials and methods

### Study design and patient population

To assess the prevalence of unfavorable lifestyle and psychosocial factors, a multicenter cross-sectional study was conducted. Patients with IBD were enrolled from an academic and a non-academic hospital in the southern region of the Netherlands (Maastricht University Medical Centre+ and Zuyderland Medical Centre). The study included patients who used the validated telemedicine platform myIBDcoach between January 1 and October 31, 2022. During this period, the platform had approximately 1300 active users across the participating hospitals [21]. This telemedicine system is part of routine clinical care for remote monitoring and is therefore covered by health insurance. Patients using myIBDcoach complete various questionnaires as part of routine clinical care at intervals ranging from one to four months, depending on disease activity and patient preference. Additionally, all patients visit the outpatient clinic at least once a year. The questionnaires cover aspects such as disease activity, lifestyle factors, and psychosocial functioning. All were administered in Dutch.

To gather patients’ perspectives on unfavorable lifestyle and psychosocial factors, a nationwide survey was developed. This was a collaboration among representatives of the Dutch IBD patient organization Crohn & Colitis NL (MS, DvdH), psychologists (QB, MS), a nutritional expert (DJ), an IBD researcher (ARA), and senior IBD specialists from 3 large non-academic (TR, AvB, RW) and 2 academic hospitals (MP, ZM, GD). Potential survey items originated from an extensive literature review and the validated Groningen IBD Environmental Questionnaire (GIEQ) [22]. During an online consensus meeting, lifestyle and psychosocial factors were prioritized. A second meeting was held to develop questions to assess the patient’s perspective regarding the impact on intestinal symptoms, motivation to change, and perception of support received. All items were formulated at B1 Dutch language level according to language guidelines (*i.e.*, understandable for the vast majority of the Dutch population). The nationwide survey was conducted online from April 7 to May 1, 2022. It was distributed to 2192 members of the Crohn & Colitis NL patient panel, all of whom had provided consent to participate in surveys. Of these, 637 individuals (29.1%) completed the survey, accounting for 55.5% of all respondents to the nationwide survey (n = 1148). The remaining half of the study population was reached through the newsletter and social media channels of Crohn & Colitis NL, the telemedicine platform myIBDcoach, and the IBDREAM registry [21,23]. Respondents voluntarily filled out the self-administered questionnaire, with the prerequisite that they were 18 years or older.

### Data collection

For users of the telemedicine platform myIBDcoach, demographic data were extracted from electronic patient records. These included sex, age, age at diagnosis, comorbidities (Charlson Comorbidity Index), disease entity, disease duration, and Montreal classification at diagnosis. IBD was diagnosed by certified gastroenterologists using the international diagnostic criteria of the European Crohn’s and Colitis Organization (ECCO) and confirmed by endoscopic, radiological, and/or histological findings [24,25]. Baseline characteristics, such as body mass index (BMI), and patient-reported outcomes on clinical disease activity, lifestyle, and psychosocial factors were collected via the telemedicine platform (S1 Table). For patients with multiple measurements available, the one closest to the national survey distribution period, either before or after, was selected. Clinical disease activity was assessed using the Monitor IBD At Home (MIAH) questionnaire.. This questionnaire includes patient-reported symptoms such as rectal bleeding, mucus loss, stool frequency, urgency, fatigue, and abdominal pain [26]. Nutritional assessment included self-reported adherence to a specific diet and perceived dietary impact on disease and/or quality of life. Patients who reported a dietary impact completed the Food-related quality of life questionnaire (FR-QoL-29; score range: 29–145, higher scores indicating better food-related quality of life), which was validated for use in IBD [27]. Smoking status (current and prior) and alcohol consumption, assessed through patient-reported weekly intake, were recorded. In line with recommendations from the Health Council of the Netherlands and the World Cancer Research Foundation, excessive alcohol consumption was defined as >7 units per week [28]. Physical activity was assessed by the number of days patients engaged in ≥30 minutes of moderate-intensity exercise (*e.g.*, brisk walking, cycling) in the past week. This aligned with the Dutch healthy exercise norm (“Nederlandse Norm Gezond Bewegen”) recommending this for at least five days per week [29]. Cardiorespiratory fitness, or functional capacity, was estimated using the 4-question Modified Duke Activity Status Index (M-DASI-4Q) [30,31]. While no established cut-off values exist for IBD patients, previous research indicated that a score ≤2 points correspond to a 52.0% probability of achieving an oxygen uptake at peak exercise (VO_2_peak) of >16 ml/kg/min as assessed with gold standard objective exercise testing. As individuals with VO_2_peak below this threshold may have an increased risk of health issues [31], patients with a score of ≤2 points were classified as low estimated cardiorespiratory fitness. Sleep, stress, and emotional distress were evaluated using brief screening questions based on instruments validated in chronic conditions, including IBD [32–34]. Sleep quality was assessed using a 5-point Likert scale on poor sleep frequency, ranging from 1 (rarely or never) to 5 (most of the time or always). Perceived stress was measured on a 10-point Numeric Rating Scale (NRS), ranging from 1 (no stress) to 10 (extreme stress), with a cut-off of 4 distinguishing low and high perceived stress levels based on expert consensus [6]. Emotional distress was assessed using a 5-point Likert scale for feelings of sadness, anxiety, frustration, shame, or other unpleasant emotions, ranging from 1 (rarely or never) to 5 (most of the time or always), and a single screening question, from the validated IBD-control questionnaire for anxiety and depression related to bowel disease [34].

For the nationwide survey cohort, respondents anonymously reported baseline characteristics, including sex, age, disease entity, and disease duration. Those without IBD (n = 9) or with missing disease phenotype data (n = 13) were excluded from further analysis. Respondents answered three closed-ended and three open-ended questions on lifestyle and psychosocial factors. Factors included unhealthy diet, smoking, alcohol, physical inactivity, poor sleep, stress, symptoms of anxiety and depression, and lack of social support (S2 Table). For each factor, respondents were asked whether they believed it affected intestinal symptoms and if they were motivated to make changes. Those already taking action were asked to describe their efforts. Additionally, respondents were asked in an open question about their need for help in changing lifestyle or psychosocial factors. Finally, they were asked whether they had received or desired support from healthcare professionals of the hospital and their satisfaction with the support.

### Data analysis

All statistical analyses were performed using IBM SPSS 27.0 software. Demographic and clinical characteristics, as well as lifestyle and psychosocial factors of patients of the telemedicine study population, were presented as numbers and percentages (%) for categorical variables and as means with standard deviations (SD) or medians with interquartile ranges (IQR) for continuous variables, depending on data distribution. Data was presented separately for Crohn’s disease (CD) and ulcerative colitis (UC), the latter also including patients with IBD-unclassified (IBD-U).

Data from the nationwide survey were reported descriptively as percentages (%) of specific responses to closed questions. Additionally, all responses to open-ended questions regarding each lifestyle or psychosocial factor were thematically analyzed and categorized.

### Ethical considerations

The medical research ethics committee of the Maastricht University Medical Centre granted approval for the reutilization of data obtained from routine care captured through the telemedicine platform myIBDcoach and the electronic patient record from the hospital for scientific research purposes (METC 2019−1115). All patients provided written informed consent. Participants of the nationwide survey provided consent for the use of their responses for scientific research.

## Results

### Telemedicine platform

The telemedicine study population consisted of 460 IBD patients with a mean age of 51.8 years, of which 244 (53.0%) were diagnosed with CD, 213 (46.3%) with UC, and 3 with IBD-U (0.7%). All demographic and clinical characteristics are presented in Table 1.

**Table 1:** Demographic and clinical characteristics of patients with IBD from the telemedicine platform and the nationwide survey.

	Telemedicine cohort (*n* = 460)			Nationwide survey cohort (*n* = 1126)		
	*Total (n = 460)*	*CD (n = 244)*	*UC (n = 216)* ^a^	*Total (n = 1126)* ^b^	*CD (n = 610)*	*UC (n = 516)* ^c^
Age, *mean (SD)*	51.8 (14.4)	49.9 (14.6)	54.0 (13.9)	50.7 (15.4)	49.4 (15.2)	52.2 (15.6)
Sex, female, *n (%)*	251 (54.6)	145 (59.4)	106 (49.1)	772 (68.6)	442 (72.6)	330 (64.0)
Disease duration						
in years, *median (IQR)*	12.3 (13.9)	12.3 (14.1	12.4 (12.3)			
<1 year, *n (%)*				61 (5.4)	33 (5.4)	28 (5.4)
1 to 2 years, *n (%)*				61 (5.4)	35 (5.7)	26 (5.0)
2 to 5 years, *n (%)*				134 (11.9)	66 (10.8)	68 (13.2)
5 to 10 years, *n (%)*				192 (17.1)	97 (15.9)	95 (18.4)
>10 years, *n (%)*				677 (60.2)	378 (62.1)	299 (57.9)
BMI (kg/m^2^), *median (IQR)*	25.7 (5.4)	25.7 (5.7)	25.6 (5.3)			
Charlson comorbidity index, *n (%)*						
0	183 (39.8)	106 (43.4)	77 (35.6)			
1-2	190 (41.3)	97 (39.8)	93 (43.1)			
>2	87 (18.9)	41 (16.8)	46 (21.3)			
Clinical disease activity^d^^,^ ^e^, *n (%)*						
Remission	346 (75.5)	159 (65.7)	187 (86.6)			
Active disease	112 (24.5)	83 (34.3)	29 (13.4)			
Age at diagnosis, *median (IQR)*	35.0 (23.2)	30.7 (22.6)	38.6 (21.8)			
Age at diagnosis^f^, *n (%)*						
< 17 years	16 (3.5)	10 (4.1)	6 (2.8)			
17-40 years	259 (56.3)	153 (62.7)	106 (49.1)			
> 40 years	185 (40.2)	81 (33.2)	104 (48.1)			
Disease extent at diagnosis^f^, *n (%)*						
E1: proctitis			68 (31.5)			
E2: left-sided colitis			93 (43.1)			
E3: pancolitis			55 (25.5)			
Disease location at diagnosis^f^, *n (%)*						
L1: ileal		87 (35.7)				
L2: colonic		82 (33.6)				
L3: ileocolonic		75 (30.7)				
+ Upper GI disease		8 (3.3)				
Disease behavior at diagnosis^f^, *n (%)*						
B1: non-stricturing, non-penetrating		207 (84.8)				
B2: stricturing		25 (10.2)				
B3: penetrating		12 (4.9)				
+ Perianal disease		21 (8.6)				

Abbreviations: BMI = body mass index, CD = Crohn’s disease, GI = gastrointestinal, IQR = interquartile range, SD = standard deviation, UC = ulcerative colitis.

^a^Of which 3 were diagnosed with IBD unclassified (IBD-U);

^b^Patient characteristics were missing for few individuals (i.e., sex for n = 1, age for n = 8, and disease duration for n = 1);

^c^Of which 24 were diagnosed with IBD unclassified (IBD-U);

^d^According to the Monitor IBD At Home Questionnaire (MIAH);

^e^Based on a total of n = 458 (CD, n = 242; UC and IBD-U, n = 216);

^f^According to the Montreal Classification.

An overview of patient-reported lifestyle and psychosocial factors is displayed in Table 2. In the telemedicine outpatient cohort, about half of the patients (50.7%) believed that diet has an impact on the disease or quality of life, and the mean food-related quality of life score for patients who completed the questionnaire was 103.6. Further, 16.3% of patients reported adhering to a specific diet, primarily motivated by the desire to lose weight (44.0%) or to manage their IBD for various reasons (32.0%). Of all patients, 9.3% were currently smoking, and 8.0% consumed more than 7 units of alcohol per week. Most patients (67.4%) indicated they engaged in moderate-intensity physical activity less than 5 days a week. Regarding estimated cardiorespiratory fitness, 40.7% of patients scored only 2 or less out of 4 points on the screening tool M-DASI-4Q. Further, approximately one-third of the study population reported a poor night of sleep at least regularly in the past week. Furthermore, 37% of the study population reported high perceived stress levels (NRS ≥ 4), and about 34% reported feelings of emotional distress at least occasionally. Only a small percentage (2.5%) of patients reported anxious or depressive symptoms because of their bowel disease.

**Table 2:** Lifestyle and psychosocial factors of IBD patients using telemedicine platform.

	Total (*n *= 460)	CD (*n* = 244)	UC (*n *= 216)^a^
Current diet, *n (%)*			
No specific diet	385 (83.7)	199 (81.6)	186 (86.1)
Yes, for my IBD	24 (5.2)	16 (6.6)	8 (3.7)
Yes, for another intestinal disorder	6 (1.3)	3 (1.2)	3 (1.4)
Yes, for weight loss	33 (7.2)	19 (7.8)	14 (6.5)
Yes, for weight gain	5 (1.1)	3 (1.2)	2 (0.9)
Yes, for another reason	7 (1.5)	4 (1.6)	3 (1.4)
Impact of diet on disease/quality of life, yes, *n (%)*	233 (50.7)	138 (56.6)	95 (44.0)
Food related quality of life score^b^, *mean (SD)*	103.6 (20.9)	97.7 (20.9)	111.4 (18.6)
Smoking status, *n (%)*			
Current smoker	43 (9.3)	32 (13.1)	11 (5.1)
Ex-smoker	221 (48.0)	111 (45.5)	110 (50.9)
Never smoked	196 (42.6)	101 (41.4)	95 (44.0)
Alcohol consumption, *n (%)*			
0 units/week	194 (42.2)	117 (48.0)	77 (35.6)
1-7 units/week	229 (49.8)	108 (44.3)	121 (56.0)
>7 units/week	37 (8.0)	19 (7.8)	18 (8.3)
Moderate-intensity physical activity for ≥ 30 minutes, *n (%)*			
0 days a week	53 (11.5)	25 (10.2)	28 (13.0)
1-2 days a week	118 (25.7)	69 (28.3)	49 (22.7)
3-4 days a week	139 (30.2)	76 (31.1)	63 (29.2)
5 or more days a week	150 (32.6)	74 (30.3)	76 (35.2)
Estimated cardiorespiratory fitness^c^^,^^d^, *n (%)*			
0 points	42 (12.3)	21 (11.2)	21 (13.5)
1 point	46 (13.5)	26 (13.9)	20 (12.9)
2 points	51 (14.9)	28 (15.0)	23 (14.8)
3 points	74 (21.6)	41 (21.9)	33 (21.3)
4 points	129 (37.7)	71 (38.0)	58 (37.4)
Poor night’s sleep, *n (%)*			
Rarely or never	162 (35.2)	83 (34.0)	79 (36.6)
Occasionally	142 (30.9)	72 (29.5)	70 (32.4)
Regularly	88 (19.1)	52 (21.3)	36 (16.7)
Often	37 (8.0)	21 (8.6)	16 (7.4)
Most of the time or always	31 (6.7)	16 (6.6)	15 (6.9)
Perceived stress score (NRS 1–10)*, median (IQR)*	3 (4)	3 (4)	2 (4)
High perceived stress (NRS ≥ 4), *n (%)*	169 (36.7)	98 (40.2)	71 (32.9)
Feelings of emotional distress, *n (%)*			
Rarely or never	304 (66.1)	156 (63.9)	148 (68.5)
Occasionally	90 (19.6)	57 (23.4)	33 (15.3)
Regularly	47 (10.2)	23 (9.4)	24 (11.1)
Often	12 (2.6)	5 (2.0)	7 (3.2)
Most of the time or always	7 (1.5)	3 (1.2)	4 (1.9)
Anxious or depressive symptoms because of bowel disease, *n* (%)	12 (2.6)	6 (2.5)	6 (2.8)

Abbreviations: CD = Crohn’s disease, IBD = inflammatory bowel disease, IQR = interquartile range, NRS = numeric rating scale, SD = standard deviation, UC = ulcerative colitis.

^a^Of which 3 were diagnosed with IBD unclassified (IBD-U);

^b^Based on a total of **n* *= 63 (CD, **n* *= 36; UC and IBD-U, **n* *= 27);

^c^Total score on Modified Duke Activity Status Index (M-DASI-4Q);

^d^Based on a total of **n* *= 342 (CD, **n* *= 187; UC and IBD-U, **n* *= 155).

### Nationwide survey

The Dutch nationwide survey, which evaluated the patient’s perspective regarding various lifestyle and psychosocial factors, was completed by 1126 individuals, whose demographic and clinical characteristics are presented in Table 1.

**Fig 1:**
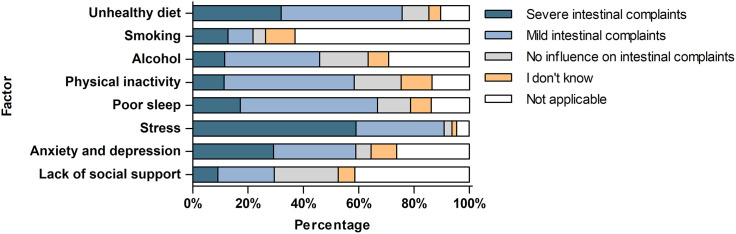
Patients’ perspective on the impact of lifestyle and psychosocial factors on intestinal symptoms.

The patients’ perspectives on the impact of lifestyle and psychosocial factors on intestinal symptoms are presented in Fig 1. The majority of patients thought that stress, unhealthy diet, poor sleep, physical inactivity, and anxiety or depression could lead to intestinal symptoms. Almost all patients (90.9%) thought that stress could lead to mild (31.9%) or even severe (59.0%) intestinal symptoms. The majority of patients responded that an unhealthy diet and poor sleep could cause mild or severe intestinal symptoms (75.7% and 66.8% of patients, respectively). Nearly 60% of patients thought that physical inactivity, as well as symptoms of anxiety and depression, could have an impact on intestinal symptoms. Concerning alcohol, nearly half of the patients (45.9%) thought it might contribute to intestinal symptoms. Smoking was thought to be associated with intestinal symptoms in about one in five (21.8%), whereas the lack of social support was identified as a culprit in about one in three (29.5%).

**Fig 2:**
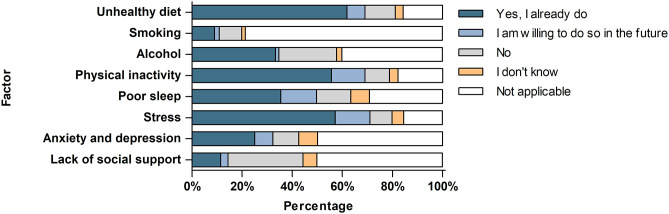
Current action or motivation to change lifestyle and psychosocial factors.

**Fig 3:**
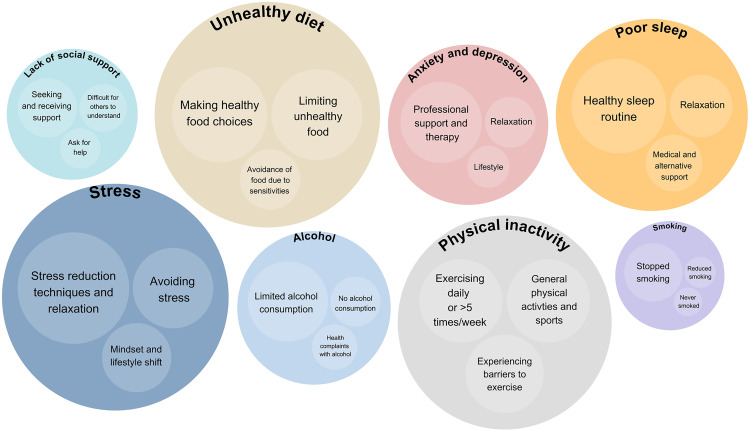
Thematic categorization of responses to the open-ended question *‘Could you give a description of actions already taken?’*, presented per lifestyle or psychosocial factor.

Regarding current actions or motivation to change lifestyle and psychosocial factors, around 70% of patients indicated they were already taking action or motivated to do so, particularly in relation to diet, stress management, and physical activity (Fig 2). Qualitative responses to the open-ended question about actions already taken revealed that dietary changes most often included making healthier food choices, limiting unhealthy foods, and, in some cases, avoiding specific foods due to sensitivities. Fig 3 presents the most frequently mentioned themes for each lifestyle and psychosocial factor, with a detailed thematic overview provided in S3 Table. In managing stress, patients described engaging in relaxation techniques, actively avoiding stressors, and pursuing a broader mindset and lifestyle shift. For physical activity, patients described exercising daily or more than five times per week or engaging in general physical activities and sports, though barriers to exercise were also acknowledged. Nearly 50% of all respondents indicated they were already taking action or were willing to do so in the future with regard to poor sleep. Actions taken in this area included adopting a healthy sleep routine, practicing relaxation, and seeking medical or alternative support. When addressing symptoms of anxiety and depression, 32.2% of respondents reported current or intended efforts. These included seeking professional support and therapy, incorporating relaxation techniques, and implementing broader lifestyle changes. Likewise, 33.3% of participants reported having taken steps regarding their alcohol consumption, typically through limiting intake, abstaining, or in response to alcohol-related complaints. Only 14.4% of patients reported current or intended actions related to a lack of social support. Among these, common responses included seeking help, asking for support, and acknowledging that others may find it difficult to understand their situation. Finally, 10.9% of patients expressed willingness to change their smoking behavior. Among those who had taken action, patients reported quitting, reducing smoking, or indicated that they had never smoked. However, as the survey did not assess smoking prevalence within this cohort, the number of potential quitters could not be determined.

**Fig 4:**
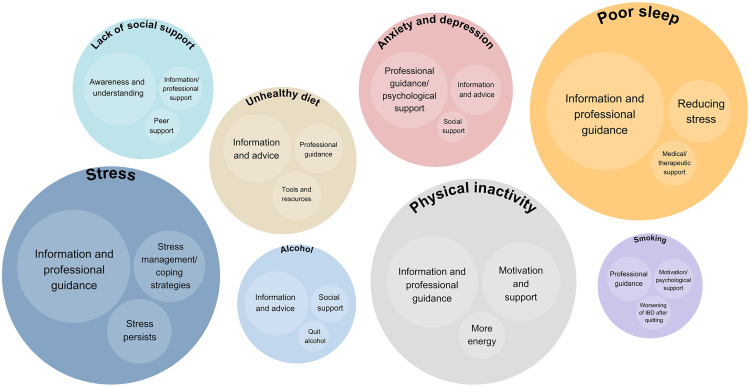
Thematic categorization of responses to the open-ended question *‘What do you need that could help you make a* change?’, presented per lifestyle or psychosocial factor.

Several key needs to facilitate changes in lifestyle and psychosocial factors were identified from the open-ended responses, as illustrated in Fig 4. A comprehensive overview is provided in S3 Table. Across all domains, one of the most frequently reported need was access to information and professional guidance. For stress, additional needs included coping strategies, although some noted that stress persisted despite efforts. In relation to physical inactivity, patients also emphasized the need for motivation, support, and increased energy. For poor sleep, patients sought guidance, stress reduction, and medical or therapeutic support. In improving diet, participants highlighted the need for dietary advice, practical tools, and resources. Those addressing anxiety and depression reported needing psychological support, information, and social support. Similarly, needs related to alcohol reduction included information, peer support, and help to quit alcohol. Patients struggling with lack of social support expressed the need for awareness, peer support, and professional help. For smoking, support needs focused on psychological guidance and concern over IBD symptom worsening after quitting.

**Fig 5:**
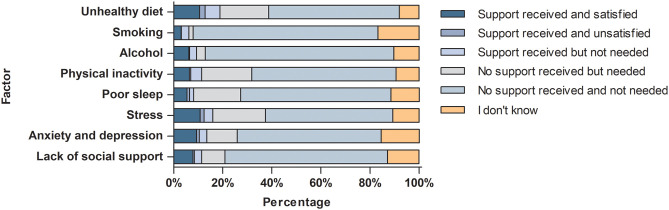
Support received for lifestyle and psychosocial factors from healthcare professionals of the hospital and satisfaction with this support.

Overall, less than a fifth of all respondents reported receiving support for various lifestyle and psychosocial factors from healthcare providers from the hospital (Fig 5). The lowest percentage (6.2%) was observed for smoking and the highest (18.9%) for unhealthy diet. The majority of these patients (48.4–69.3%) were satisfied with the support received. Approximately one-fifth of respondents reported unmet needs for hospital-based support regarding stress (21.4%), physical inactivity (20.4%), unhealthy diet (19.8%), and poor sleep (19.2%). These numbers were even higher among the subgroup of respondents who were either already addressing or motivated to address various lifestyle and psychosocial factors in the future (S1 Fig). Within this subgroup, a considerable proportion of respondents reported that they desire support from the hospital in various areas, including lack of social support (33.3%), poor sleep (32.0%), anxiety or depression (29.9%), stress management (27.2%), physical inactivity (26.1%), and diet (25.4%), yet did not receive it. Approximately half to three-quarters of the total population reported that no support was offered for various lifestyle and psychosocial factors and that they did not require any support, ranging from 51.9% for stress to 76.8% for alcohol.

Although hospital-based support for lifestyle and psychosocial factors among IBD patients was limited, many respondents described seeking help elsewhere. Open-ended responses revealed that patients often turned to primary care providers, dietitians, mental health professionals, or physiotherapists outside hospital settings. Many relied on self-management strategies, such as dietary adjustments, stress reduction techniques, online information seeking, and use of health apps. However, patients often highlighted a lack of clear, personalized, and trustworthy guidance, especially regarding nutrition, mental health, sleep, and stress. Common needs included concrete advice, continued professional support, motivation, and help overcoming practical barriers.

S2–S4 Figs provide an overview of patients’ perspectives on lifestyle and psychosocial factors, categorized by Crohn’s disease and ulcerative colitis.

## Discussion

In this study, we provide supporting evidence that various unfavorable disease-modifying lifestyle and psychosocial factors occur frequently in individuals with IBD. Most patients thought that these factors increase the burden of IBD symptoms and were already implementing or motivated to implement changes regarding various factors. Most important, about a fifth of the population reported a perceived lack of support offered by healthcare professionals despite expressing a need for it.

About half of the telemonitoring study population thought that diet affected disease or quality of life, with a mean food-related quality of life score of 103.6. Although no validated cutoff value exists, this score exceeded that of the questionnaire’s validation cohort (89.5), yet remained below the mean score observed in a healthy volunteer cohort (123.0) within the same study [27]. These findings are consistent with the nationwide survey findings, which showed that most patients associated consuming unhealthy foods with intestinal symptoms. However, our survey focused on ‘unhealthy foods’, while previous research indicates that even healthy foods, like high-fiber products and (raw) vegetables, can trigger symptoms in IBD patients [35]. This suggests that our findings may underestimate the impact of diet on intestinal symptoms. These dietary triggers may lead to avoidance behaviors, providing short-term relief but potentially causing dietary deficiencies in several micronutrients, such as iron, folic acid, zinc, magnesium, calcium, vitamins A, B12, D, E, and K [2,19,36–38]. Further, the majority of patients in the routine care telemedicine study population did not follow any specific diet. Several dietary patterns are commonly recommended for patients with IBD, including the Mediterranean diet, Specific Carbohydrate Diet, and Crohn’s Disease Exclusion Diet. Recommendations also include increasing the intake of fruits and vegetables (in the absence of strictures) in Crohn’s disease and avoiding additives, processed foods, and red or processed meat in ulcerative colitis [39–41]. Unfortunately, detailed information on whether patients followed such dietary approaches was not collected within the platform. Additionally, it is important to acknowledge that the question about diet in the telemedicine platform did not include any measurement regarding the quality of the regular diet and did not differentiate between a 'healthy' or an 'unhealthy' diet. It is also possible that people made changes to their eating habits, such as cutting back on certain food groups, without considering these adjustments as following a specific diet. The variability in dietary triggers among individuals with IBD underscores the need for personalized dietary advice that considers individual responses. Therefore, we are currently working on integrating dietary analysis and advice into the telemonitoring platform.

Only one-third of the study population met the former Dutch exercise norm of at least 30 minutes of moderate-intensity physical activity per day, 5 days a week. The current norm recommends 150 minutes per week, yet only 44% of the Dutch population met this guideline in 2022 [42]. Physical inactivity, along with other potential issues like malnutrition and corticosteroid use, may likely induce unfavorable physical fitness in patients with IBD, as indicated by the relatively high prevalence of reduced estimated cardiorespiratory fitness in our telemedicine study population [43–46]. Additionally, 55% of our telemedicine study population was classified as overweight, with 17% being obese. These rates are comparable to other IBD cohorts, as well as the Dutch adult population, and may partly be explained by the observed physical inactivity [7,47,48]. Most respondents in the nationwide survey recognized that physical inactivity has an impact on intestinal symptoms, which implies there is a difference between patients’ knowledge and their actual behavior. Challenges like fatigue, abdominal discomfort, and unpredictable bowel movements may hinder increased physical activity in IBD patients [3,49]. While previous research suggests a positive relationship between increased levels of physical activity or exercise and outcomes in IBD patients, evidence regarding its effectiveness as a standardized treatment modality for this specific population remains limited and warrants more investigation [50,51].

Approximately 10% of the studied patients with IBD were active smokers, which is lower than the smoking rate (20.6%) in the general population in the Netherlands in 2021 [52]. This could reflect patients’ awareness of the negative impact of smoking on their disease, possibly due to medical advice received or effective smoking cessation strategies. In our telemedicine cohort, 8% reported excessive alcohol consumption (**i.e.,* *> 7 units/week), while the vast majority (92%) consumed less. This proportion exceeds the 44% of the Dutch adult population in 2022 who adhered to the advice of the Health Council (*i.e.,* abstaining from alcohol or limiting intake to one glass daily) [53]. A potential explanation for this finding could be that patients with IBD often voluntarily avoid alcohol because of subjective worsening of gastrointestinal symptoms. This is in line with findings from the nationwide survey in which it appeared that 46% of respondents believed that alcohol could lead to intestinal symptoms [7,54].

The prevalence of poor sleep (34%) in our IBD cohort is consistent with previous studies reporting frequent sleep disturbances in individuals with IBD [10]. This rate is slightly higher than in the general Dutch adult population, where approximately 25% report considerable difficulties with sleep [55]. Additionally, high perceived stress was reported by 37% of the telemedicine study population, exceeding the 21% rate in the general Dutch adult population [56]. Over 90% of surveyed IBD patients linked stress to intestinal symptoms, with over half associating it with severe symptoms, suggesting an important link with disease burden [57]. Approximately one-third of the study population reported feelings of emotional distress (*i.e.,* sadness, anxiety, frustration, shame, or other unpleasant emotions) at least occasionally in the past week, aligning with previously reported prevalences of depressive symptoms (21.0–25.2%) and anxiety (19.1–32.1%) in IBD patients [58]. However, the broad nature of the question in the telemedicine platform made direct comparisons with existing literature challenging. Notably, only 2.5% of the patients from the telemedicine cohort attributed their depressive or anxiety symptoms directly to their disease, reflecting the multifaceted nature of mental health conditions like depression and anxiety. A bidirectional relationship seems to appear between the disease course of IBD and mental health, influenced by brain-gut interactions [59]. Several interventional programs for psychological factors, such as Cognitive Behavioral Therapy (CBT), Acceptance and Commitment Therapy (ACT), hypnosis, and mindfulness, are proven effective in IBD and can be implemented in multidisciplinary care pathways (collaborating with, *e.g.*,** psychologists, social workers, IBD nurses) and telehealth interventions [60].

Many patients were proactively managing or considering managing various lifestyle and psychosocial factors, showing a positive shift toward health-conscious behavior. However, about one-fifth of respondents to the nationwide survey felt their needs for support in managing diet, stress, physical activity, and sleep quality were unmet. Among those actively making changes or motivated to do so, 25% to 34% reported a lack of support despite needing it. A perceived lack of support from hospital-based healthcare professionals may lead patients to rely on self-initiated strategies or seek help sources such as community support groups, online health forums, alternative medicine, or friends and family [19]. This highlights a noteworthy area of concern in healthcare delivery, underscoring the necessity for focused attention on understanding IBD patients’ specific needs, the barriers they face in accessing support, and facilitators that match their preferences. Additionally, there are opportunities for healthcare professionals beyond the hospital setting, such as primary care providers and allied healthcare professionals, to play a more active role in managing lifestyle and psychosocial factors in IBD [61,62]. Our findings show that patients often seek support from dietitians and general practitioners, particularly for dietary guidance, mental health concerns, and behavior change. Dietitians, especially those with IBD expertise, can offer personalized advice and practical tools, while primary care providers are well placed to support mental well-being and coordinate referrals. Integrating these professionals more systematically into IBD care, *e.g.,* through shared care models, better communication, and targeted training, could help address the current support gap and improve patient outcomes.

This study highlights the importance of targeting interventions towards patients who are motivated to change their behavior to ensure that resources are used efficiently and increase the likelihood of interventions being effective. While 10–15% of the population was not motivated to change most factors, a larger proportion was unwilling to change alcohol and social support habits (22% and 30%, respectively). It remains unclear from these data whether this reflects a satisfactory lifestyle and psychosocial status, limited awareness of the potential health benefits of change, or a lack of resources to support such change. This warrants further investigation in order to enhance educational initiatives and the dissemination of information as key strategies to enhance awareness and foster willingness to change. A substantial proportion of patients did not report needing or receiving support with regard to most factors, suggesting they either have adequate self-management strategies or do not recognize the need for external support [63]. Systematic assessment of lifestyle and psychosocial factors through telemonitoring platforms could help identify patient needs early and provide feedback and educational strategies as needed. Furthermore, telemonitoring platforms such as *myIBDcoach* offer a promising and accessible infrastructure for implementing and evaluating personalized lifestyle and psychosocial interventions in routine care. Future research should explore the effectiveness of such interventions, (*e.g.,* dietary counseling, stress-reduction modules, physical activity programs, sleep optimization strategies, psychological support) on disease course, symptom burden, and quality of life in patients with IBD. Also, the integration of digital biomarkers, such as step counts, heart rate, sleep duration, and activity patterns, collected passively via widely used wearable devices (*e.g.,* smartphones and smartwatches), holds significant potential for objective, continuous monitoring of lifestyle behaviors. These data may enable the early detection of unfavorable trends, such as physical inactivity or poor sleep, and allow healthcare providers to intervene proactively, thereby facilitating more personalized and preventive IBD management.

An important strength of this study was the multifaceted data collection strategy, utilizing the telemedicine platform myIBDcoach and a nationwide survey with the Dutch IBD patient organization. Furthermore, this study encompassed large sample sizes, enhancing the generalizability of its findings. However, this study also has some limitations. Firstly, it is important to acknowledge potential selection bias. The willingness of patients to engage with telemonitoring tools or participate in the patient panel of the Dutch patient organization and/or complete surveys may indicate a higher level of engagement in their disease management. Moreover, a review showed that eHealth tools are often underutilized by individuals with a lower socioeconomic position [64]. This suggests that our findings may underestimate the occurrence of unfavorable lifestyle and psychosocial factors in the broader IBD population. Second, the study’s prevalence data was based solely on users of myIBDcoach from the southern region of the Netherlands. This may limit applicability to other regions with different demographic and cultural characteristics. However, the demographic and clinical characteristics of this study population were comparable to those of the larger, population-based IBD South-Limburg cohort [65]. This cohort includes patients from the same geographical area and hospitals, supporting the representativeness of our sample for the regional IBD population. Third, the absence of prevalence data from the nationwide survey group prevented direct comparisons between the two groups, limiting our ability to interpret patient experiences in the context of actual prevalence rates within the same cohort. Fourth, some single screening questions in the telemedicine platform were not specifically validated for IBD patients, and the lack of a dedicated question on social support hindered the understanding of its prevalence. Fifth, the absence of structured dietary intake data limits personalized nutritional guidance, so future integration of dietary assessment tools in the telemonitoring platform is needed. Additionally, this study relied on self-reported data, which may have been influenced by factors such as recall bias or interpretation of the questions. Moreover, different telemonitoring platforms may vary in terms of functionality, user interface, and overall user acceptance, also potentially influencing the accuracy of self-reported data [66]. Additionally, we primarily assessed hospital-based support for lifestyle and psychosocial factors in this study, while this support could also be provided outside the hospital setting, such as by primary care providers and allied healthcare professionals. Although these forms of support were not systematically captured in the survey, responses to the open-ended questions did provide additional insights into support received outside the hospital setting. Lastly, the study’s cross-sectional design restricted the establishment of causality between lifestyle and psychosocial factors and, for example, their impact on intestinal complaints in IBD. Longitudinal studies are essential to further elucidate these relationships and to determine the direction and extent of the influence exerted by modifiable lifestyle and psychosocial factors on objective disease outcomes and parameters of subjective well-being in patients with IBD [18]. This will support the identification of potential targets for interventions aimed at improving long-term disease management.

## Conclusion

In this study we observed that unfavorable lifestyle and psychosocial factors occur frequently in patients with IBD, highlighting the need for systematic integration of monitoring these factors into patient-centered IBD care. From the patient’s viewpoint, widespread recognition of the impact of these factors on intestinal symptoms emerged, with most indicating an openness to initiate changes for various factors. These insights emphasize the importance of the implementation of targeted interventions, which are currently not often received by patients with IBD, particularly in areas where patients indicated a need for additional support. Future longitudinal research is needed to examine the impact of systematically incorporating assessments of lifestyle and psychosocial factors into routine care to provide a foundation for personalized interventions to improve clinical outcomes and patient-reported measures of well-being.

## Supporting information

S1 Fig Support received for lifestyle and psychosocial factors from healthcare professionals of the hospital among the subgroup of respondents who were already taking action or were willing to do so in the future. (DOCX)

S2 Fig Patients’ perspective on the impact of lifestyle and psychosocial factors on intestinal symptoms for patients with Crohn’s disease (A) and ulcerative colitis (B). (DOCX)

S3 Fig Current action or motivation to change lifestyle and psychosocial factors for patients with Crohn’s disease (A) and ulcerative colitis (B). (DOCX)

S4 Fig Support received for lifestyle and psychosocial factors from healthcare professionals of the hospital and satisfaction with this support for patients with Crohn’s disease (A) and ulcerative colitis (B). (DOCX)

S1 Table Overview of lifestyle and psychosocial questions in the telemedicine platform (myIBDcoach). (DOCX)

S2 Table Overview of questions about lifestyle and psychosocial factors in the nationwide survey. (DOCX)

S3 Table Thematic categorization of responses to the open-ended questions, presented per lifestyle or psychosocial factor. (DOCX)
