# Supplementary material for: Lifestyle and psychosocial factors in inflammatory bowel disease: Prevalence, impact, motivation, and support needs
Source: PLoS One. 2025 Aug 29;20(8):e0331092. doi: 10.1371/journal.pone.0331092 (PMC12396644; doi:10.1371/journal.pone.0331092)
Supplement: S1 Fig — (DOCX) [file pone.0331092.s001.docx]

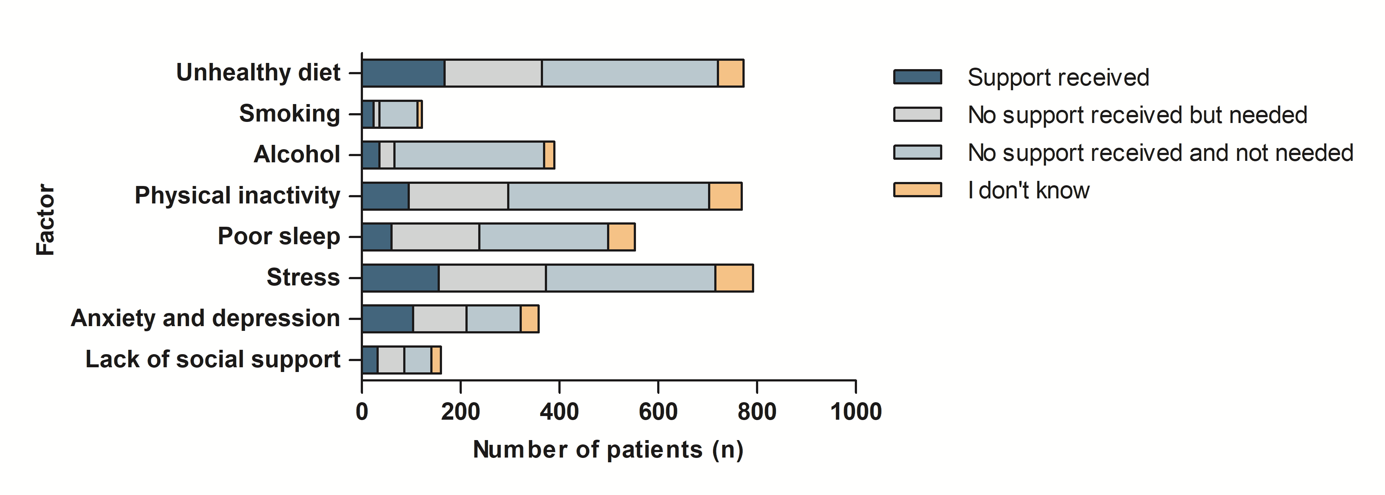


**S1 Fig. Support received for lifestyle and psychosocial factors from healthcare professionals of the hospital among the subgroup of respondents who were already taking action or were willing to do so in the future.**
