# Supplementary material for: Lifestyle and psychosocial factors in inflammatory bowel disease: Prevalence, impact, motivation, and support needs
Source: PLoS One. 2025 Aug 29;20(8):e0331092. doi: 10.1371/journal.pone.0331092 (PMC12396644; doi:10.1371/journal.pone.0331092)
Supplement: S3 Fig — (DOCX) [file pone.0331092.s003.docx]

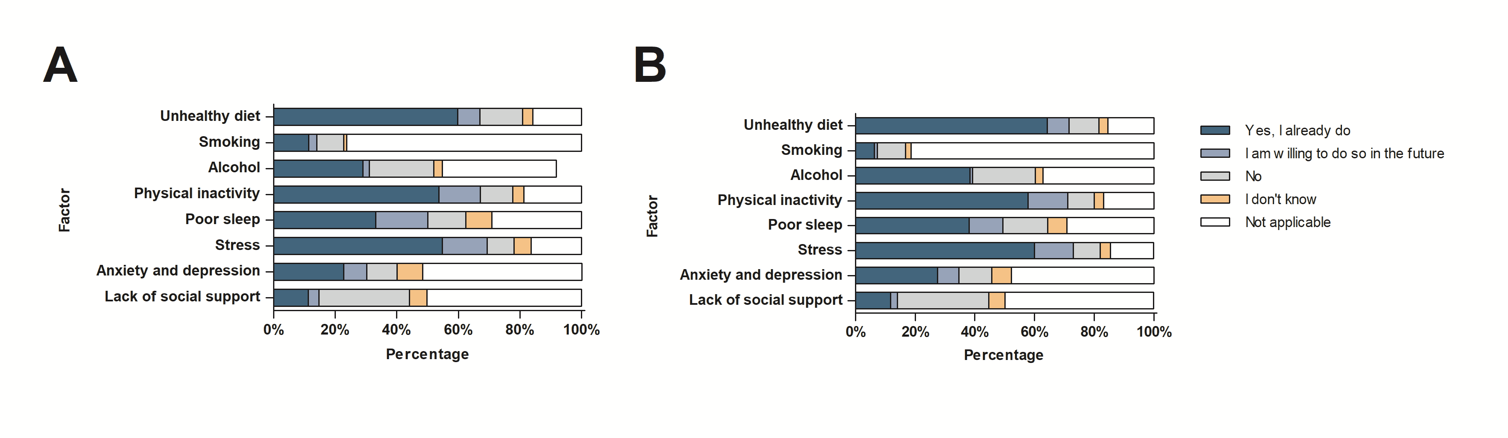


**S3 Fig.** **Current action or motivation to change lifestyle and psychosocial factors for patients with Crohn’s disease (A) and ulcerative colitis (B).**
