# Supplementary material for: Lifestyle and psychosocial factors in inflammatory bowel disease: Prevalence, impact, motivation, and support needs
Source: PLoS One. 2025 Aug 29;20(8):e0331092. doi: 10.1371/journal.pone.0331092 (PMC12396644; doi:10.1371/journal.pone.0331092)
Supplement: S1 Table — (DOCX) [file pone.0331092.s005.docx]

**S1 Table.** **Overview of lifestyle and psychosocial questions in the telemedicine platform (myIBDcoach).**

| Domain | Type | Question or questionnaire | | Scale | Answer options | |
| --- | --- | --- | --- | --- | --- | --- |
|  |  | Dutch | English |  | Dutch | English |
| Diet | Single question | “Volgt u op dit moment een dieet?” | “Are you currently following a diet?” | Catego-rical | “nee”,  “ja, om gewicht te verliezen”,  “ja, om in gewicht aan te komen”,  “ja, voor deze darmaandoening”,  “ja, voor een andere darmaandoening” | “no”,  “yes, to lose weight”,  “yes, to gain weight”,  “yes, for this intestinal condition”,  “yes, for another intestinal condition” |
|  | Single question | “Denkt u dat uw voeding invloed heeft op uw darmziekte en/of kwaliteit van leven?” | “Do you think your diet affects your bowel disease and/or quality of life?" | Dichoto-mous | “nee”,  “ja” | “no”,  “yes” |
|  | Question-naire | Food-related Quality of Life (FR-QoL-29) questionnaire  [only asked if answered ‘yes’ to the previous question] | | Points [29-145] | NA | |
| Smoking | Single question | “Rookt u?” | “Do you smoke?” | Catego-rical | “nee, ik heb nooit gerookt”,  “nee, maar ik heb wel gerookt”,  “ja” | “no, I have never smoked”,  “no, but I used to smoke”,  “yes” |
| Alcohol | Single question | “Hoeveel glazen alcohol drinkt u gemiddeld per week?” | English: “How many glasses of alcohol do you drink on average per week?" | Numeri-cal | NA | |
| Physical activity/ exercise | Single question | “In de afgelopen week, hoeveel dagen heeft u 30 minuten of meer matig intensieve lichaams-beweging gehad? Bijvoorbeeld stevig doorwandelen of harder fietsen. Het mogen ook minimaal 3 x 10 minuten zijn.” | “In the past week, how many days did you engage in at least 30 minutes of moderate-intensity physical activity? For example, brisk walking or cycling at a faster pace. It can also be at least 3 x 10 minutes.” | Catego-rical | “0 dagen”,  “1-2 dagen”,  “3-4 dagen”,  “≥ 5 dagen” | “0 days”,  “1-2 days”,  “3-4 days”,  “≥ 5 days” |
|  | Question-naire | Modified Duke Activity Status Index (M-DASI-4Q) | | Points  [0-4] | NA | |
| Sleep | Single question | “In de afgelopen week, hoe vaak had u een slechte nachtrust?” | “In the past week, how often did you have a poor night's sleep?” | Likert  [1-5] | “1. zelden of nooit”,  “2. af en toe”,  “3. regelmatig”,  “4. vaak”,  “5. meestal of altijd” | “1. rarely or never”,  “2. occasionally”,  “3. regularly”,  “4. often”,  “5. most of the time or always” |
| Perceived stress | Single question | “Als u een waarde moet geven aan uw stress op een schaal van 1 tot 10, waar 1 geen stress betekent en 10 heel veel stress, hoe zou u vandaag uw stress omschrijven?” | “If you had to rate your stress on a scale from 1 to 10, where 1 means no stress and 10 means a lot of stress, how would you describe your stress today?” | NRS  [1-10] | NA | |
| Emotional distress | Single question | “In de afgelopen week, hoe vaak had u last van somberheid, angst, frustratie, schaamte of andere vervelende gevoelens?” | “In the past week, how often did you experience sadness, anxiety, frustration, shame, or other unpleasant feelings?” | Likert  [1-5] | “1. zelden of nooit”,  “2. af en toe”,  “3. regelmatig”,  “4. vaak”,  “5. meestal of altijd” | “1. rarely or never”,  “2. occasionally”,  “3. regularly”,  “4. often”,  “5. most of the time or always” |
|  | Single question [from IBD-control] | “Heeft u in de afgelopen twee weken zich angstig of depressief gevoeld door uw darmziekte?” | “In the past two weeks, have you felt anxious or depressed because of your bowel disease?" | Catego-rical | “nee”,  “ja”,  “weet ik niet” | “no”,  “yes”,  “I don’t know” |
