# Supplementary material for: Lifestyle and psychosocial factors in inflammatory bowel disease: Prevalence, impact, motivation, and support needs
Source: PLoS One. 2025 Aug 29;20(8):e0331092. doi: 10.1371/journal.pone.0331092 (PMC12396644; doi:10.1371/journal.pone.0331092)
Supplement: S2 Table — (DOCX) [file pone.0331092.s006.docx]

**S2 Table. Overview of questions about lifestyle and psychosocial factors in the nationwide survey.**

| Domain | | Question | | Type | Answer options | |
| --- | --- | --- | --- | --- | --- | --- |
| Dutch | English | Dutch | English |  | Dutch | English |
| Leefstijl en psychosociale factoren [XXX]:  - Ongezonde voeding  - Alcohol  - Roken  - Geen/weinig bewegen  - Slechte nachtrust  - Stress  - Angst en depressie  - Gebrek aan steun van partner, familie of vrienden | Lifestyle and psychosocial factor [XXX]:  - Unhealthy diet  - Alcohol  - Smoking  - No/little physical activity  - Poor sleep  - Stress  - Anxiety and depression  - Lack of support from partner, family or friends | “Denk je dat de factor [XXX] invloed heeft op jouw darmklachten?” | “Do you think that factor [XXX] influences your bowel complaints?” | Multiple choice question (single select) | “Ja, ik denk dat ik door deze factor ernstige damklachten krijg/ kan krijgen”,  “Ja, ik denk dat ik door deze factor milde darmklachten krijg/ kan krijgen”,  “Nee, ik denk niet dat deze factor invloed heeft op mijn darmen”,  “Nee, deze factor is niet van toepassing voor mij”,  “Weet ik niet” | "Yes, I think this factor can cause severe bowel complaints”,  "Yes, I think this factor can cause mild bowel complaints”,  "No, I don't think this factor affects my intestines”,  "No, this factor does not apply to me”,  “I don't know” |
|  |  | “Wil je iets doen of veranderen aan de factor [XXX]?” | “Are you willing to take any action or make changes related to this factor [XXX]?“ | Multiple choice question (single select) | “Ja, ik wil hier in de toekomst iets aan doen”,  “Ja ik doe hier al iets aan”,  “Nee”,  “Niet van toepassing”,  “Weet ik (nog) niet” | "Yes, I am willing to take action in the future",  "Yes, I am already taking action”,  "No",  "Not applicable",  "I don't know (yet)" |
|  |  | “[XXX]: Wat heb je nodig dat jou zou kunnen helpen om dit te veranderen?” | “[XXX]: What do you need that could help you change this?” | Open question | NA | |
|  |  | “[XXX]: Kan je beschrijven wat jij hier al aan doet?” | “[XXX]: Could you give a description of these actions?” | Open question | NA | |
|  |  | “Ben je tevreden met de ondersteuning die je van je zorgverleners uit het ziekenhuis krijgt voor de factor [XXX]?” | “Are you satisfied with the support you receive from the healthcare providers from the hospital regarding factor [XXX]?” | Multiple choice question (single select) | “Ja, ik krijg ondersteuning aangeboden en ben daar tevreden over”,  “Ja, ik krijg ondersteuning aangeboden, maar ben daar niet tevreden over”,  “Ja, ik krijg ondersteuning aangeboden, maar heb hier geen behoefte aan”,  “Nee, ik krijg geen ondersteuning aangeboden, maar ik heb hier wel behoefte aan”,  “Nee, ik krijg geen ondersteuning aangeboden, maar heb hier ook geen behoefte aan”,  “Weet ik niet” | "Yes, I receive support and I am satisfied with it",  "Yes, I receive support but I am not satisfied with it",  "Yes, I receive support but do not desire it",  "No, I do not receive support but desire it",  "No, I do not receive support and do not desire it",  "I don't know" |
