# Supplementary material for: Lifestyle and psychosocial factors in inflammatory bowel disease: Prevalence, impact, motivation, and support needs
Source: PLoS One. 2025 Aug 29;20(8):e0331092. doi: 10.1371/journal.pone.0331092 (PMC12396644; doi:10.1371/journal.pone.0331092)
Supplement: S3 Table — (DOCX) [file pone.0331092.s007.docx]

**Supplementary Table 3. Thematic categorization of responses to the open-ended questions, presented per lifestyle or psychosocial factor.**

| **Question: “What do you need that could help to make changes?”** | | **Question: “Could you give a description of these actions?”** | |
| --- | --- | --- | --- |
| Answers | n | Answers |  |
| **Unhealthy diet** | | | |
| **Information and advice on nutrition**  Information and advice on healthy nutrition and diet (online)  Information and advice on nutrition and IBD (online)  Personalized advice  Concrete advice, recipes  **Professional guidance and support**  Advice and guidance from a dietitian  Advice and guidance from a HCP  Advice and guidance from a dietitian specialized in IBD  Continued attention to dietary advice  **Tools and resources**  Nutrition app  Keeping a (food) diary  Gaining experience with which food products are tolerable  **Motivation and practical support**  Help with maintaining/ motivating for a healthy diet  Lifestyle program  Guidance from a buddy  **Practical barriers and needs**  Affordable healthy food  Medication for sugar cravings  **Other**  No advice needed  Do not know | **55**  27  12  8  8  **28**  14  7  4  3  **17**  14  2  1  **10**  7  2  1  **3**  2  1  **4**  3  1 | **Making (more) healthy food choices**  Ensuring/increasing vegetable and fruit intake  Varied food intake or unspecified  Fiber intake  The Wheel of Five  Ensuring protein intake  Fish consumption  **Limiting the intake of unhealthy foods**  Limiting high-fat and/or fast-food  Limiting sugar intake  Limiting other unhealthy foods in general (e.g. snacks,  carbohydrates, chips, alcohol, caffeine)  Limiting salt intake  **Avoidance of food products based on sensitivities**  Unspecified  Specific (uncooked) vegetables or fruits  Spices  Onion and/or garlic  Cabbage  Grains, nuts, and seeds  Alcohol  Carbonic acid drinks  Coffee/caffeine  **Following a diet**  Low-lactose or lactose-free  Low-gluten or gluten-free  FODMAP diet  Unspecified  Low carbohydrates  Intermittent fasting  Crohn's Disease Exclusion Diet  Paleo diet  Specific carbohydrate diet  Tracking calories  Exorphin-free diet  Weight Watchers  **Avoidance or minimization of processed food products**  **Less or no animal products**  Less or reduced (red) meat and/or fish  Vegetarian  Vegan or plant-based  Mostly plant-based  No eggs  No shellfish  **Guidance and dietary advice**  Dietitian  Orthomolecular physician  “IBD eet je mee” program  Osteopath  Vitality coach  Psychologist for binge eating disorder  Naturopathic therapist  Information from “Voeding Leeft” foundation   Information from “Project Gezond”  Information from myIBDcoach  Information from “Voedingscentrum”  Webinar from Dutch patient organization Crohn Crolitis NL  Book from dr. Tamara de Weijer  Advice and recipes from Jasper Alblas  Lifestyle programme  Participation in scientific research on nutrition  **Awareness/acceptance of consequences unhealthy eating**  **Adjusting meal frequency or portion sizes**  Regularly eating during the day  Eating less in small portions  Eating at fixed times during the day  Intuitive eating  No eating in the evening  **Balancing healthy eating with occasional unhealthy eating**  Sometimes unhealthy eating  Alternating between healthy and unhealthy eating  Compensating by eating healthy after unhealthy eating  Unhealthy eating only in weekends  Unhealthy eating once a week  **Drinking enough water**  **Other**  Supplements  Ensuring sufficient calorie intake (e.g., by using Nutridrink)  Gastric sleeve surgery  Share knowledge with others | **331**  157  99  47  9  16  3  **303**  126  95  80  2  **133**  43  17  16  13  11  10  9  8  6  **118**  60  21  13  7  4  3  3  2  2  1  1  1  **101**  **95**  52  31  4  4  2  2  **48**  31  2  2  1  1  1  1  1  1  1  1  1  1  1  1  1  **23**  **20**  8  7  3  1  1  **20**  7  6  3  2  2  **16**  **9**  5  2  1  1 |
| Total respondents | 88 |  | 610 |
| **Smoking** | | | |
| **Professional guidance and support**  Support and guidance from a HCP  Help with quitting smoking  Quitting with a group  Admission to a stop-smoking clinic  **Motivation and psychological support**  Motivation to quit smoking and to persevere  Help with reasons for smoking, e.g. processing the past  Quit attempts failed, considering hypnosis  **Tools and resources**  Quit smoking app  Information about (quitting) smoking and its relationship with IBD (online)  **Barriers and challenges**  Worsening of IBD symptoms after quitting smoking  Smoking helps with defecation  Quit attempts failed, but smoking reduced  **Other**  No need for help with reducing/quitting smoking  Quitting smoking  Do not know | **9**  5  2  1  1  **7**  4  2  1  **3**  2  1  **6**  3  2  1  **5**  2  1  2 | **Never smoked**  **Stopped smoking**  Stopped smoking  Quit smoking because of IBD diagnosis  Quit but started again or struggle to stay quit  Had a flare after quitting smoking  Improved bowel movements after quitting smoking  **Reducing or controlling smoking**  Reducing smoking but not completely quit  Only smoking on occasions  Switched from cigarettes to cigars and vaping  Smokes periodically  Occasionally smoking a joint  **Health impact and considerations**  More IBD complaints after quitting smoking  Avoiding second-hand smoke  Smoking affects intestinal health, do not quit all at once  **Support and coping mechanisms**  Asking for support from relatives  Psychological help for feelings and habits related to smoking | **16**  **63**  54  3  2  2  2  **18**  11  3  2  1  1  **9**  6  2  1  **2**  1  1 |
| Total respondents | 23 |  | 92 |
| **Alcohol** | | | |
| **Information and (professional) guidance**  Advice and information (online)  Advice and information about alcohol in relation to IBD/ intestinal health  Advice from HCPs  Help with reasons for drinking  **Social support**  Advice on dealing with alcohol in social settings  Social support  **Tools and resources**  App  **Reduction or quitting alcohol**  No/ quitting alcohol consumption or drinking in moderation  No alcohol consumption in case of intestinal complaints  Ban of alcohol  **Other**  No advice needed  Do not know | **17**  8  6  2  1  **4**  2  2  **2**  2  **6**  4  1  1  **7**  5  2 | **No alcohol consumption**  No alcohol consumption  Quit drinking alcohol  No alcohol consumption with IBD symptoms  No alcohol consumption due to medication  Drinking other beverages, non-alcoholic drinks  No alcohol consumption at home  **Limited or controlled alcohol consumption**  Limited alcohol consumption  Drinking less alcohol, in frequency and/or quantity  Limited alcohol consumption, only on occasions  Limited alcohol consumption, only on weekends  Limited alcohol consumption, in frequency and/or quantity  Only consuming alcohol when the next day allows for IBD-  symptoms  Drinking less alcohol, only on weekends and/or occasions  Limited alcohol consumption due to medication  Only drinking alcohol on weekends or on occasions  Being mindful of alcohol consumption  Refusing alcohol more often  **Alcohol and health considerations**  More IBD symptoms due to alcohol (or feeling very tired)  No beer (and spirits), only wine  No or less spirits  Sensing when drinking alcohol is (not) possible  No mixing of different alcohol drinks  No beer (and wine), only spirits  No alcohol with preservatives or high sugar content  Alcohol has no effect on IBD symptoms  Taking a stomach protector  **Struggles or efforts to reduce alcohol consumption**  Trying to drink less alcohol but failing  Support / online program to reduce alcohol consumption  **Increased alcohol consumption**  Drinking more alcohol  Alcohol brings relaxation | **97**  50  25  16  3  2  1  **250**  100  71  21  19  11  10  8  5  3  1  1  **45**  28  4  3  2  2  2  2  1  1  **4**  2  2  **2**  1  1 |
| Total respondents | 32 |  | 345 |
| **Physical inactivity** | | | |
| **Information and professional guidance**  Guidance/ coaching by a physiotherapist/ trainer  Information and advice (online)  Advice/ help from a HCP  Guidance in exercising and fatigue  Advice from a HCP on exercising with IBD  Advice on exercising with a stoma  **Motivation and support**  Motivation and perseverance  App  Exercising in a group / with a buddy  Exercise program (personalized)  **Barriers to exercising**  More (free) time to exercise  Unable to exercise due to comorbidity  **IBD and exercising**  Need more energy to exercise  Exercising less or not at all due to IBD symptoms  Limited in exercising due to needing to go to the toilet  Exercise causes more bowel complaints  **Financial support**  Reimbursement of costs from health insurance  High costs for some sports  **Other**  Started exercising or already exercising  Do not know | **76**  26  23  14  7  5  1  **56**  26  15  13  2  **13**  7  6  **31**  20  6  3  2  **3**  2  1  **13**  7  6 | **High-frequency exercisers (daily or 5+ times per week)**  Walking and/or cycling (daily or frequently)  Exercise, fitness or strength training (daily)  Sports (5+ times per week)  Gardening or active work (daily)  Monitoring movement, setting personal daily goal(s)  Participating in “Nederland in Beweging” (TV program) (daily)  Swimming, yoga, or dancing (daily)  **Medium-frequency exercisers (3-4 times per week)**  Sports (*e.g.* running, football, volleyball, golf) (3-4x per week)  Walking and/or cycling (3-4x per week)  Exercise, yoga (3-4x per week)  Fitness, strength training (3x per week)  **Low-frequency exercisers (1-2 times per week)**  Sports (*e.g.* running, horse riding, tennis, football) (1-2x per  week)  Fitness, cardio- and/or strength training (1-2x per week)  Swimming, aquafit (1-2x per week)  Walking and/or cycling (1-2x per week)  Dancing, ballet (1x per week)  Participating in “Nederland in Beweging” (TV program),  exercises via app, jeu de boules (1x per week)  Yoga (1x per week)  **General physical activities and sports**  Walking and cycling  Sports, exercise  Household activities (grocery shopping, gardening, playing  with grandchild), taking stairs  Fitness, strength training, body pump, HIIT training, boxing  Yoga, pilates, meditation, tai chi  Swimming, aqua fitness  Running, football, volleyball  Participating in “Nederland in Beweging” (TV program)  Tennis, badminton, golf  Dancing, ballet  Iceskating, rollerblading, trampoline  Physiotherapy exercises  **Impact of movement on health and limitations**  Positive impact of exercise on IBD symptoms, disease  course, bowel movements, prevents flares  Exercise benefits well-being, energy levels, physical/ mental  health  More IBD symptoms due to exercise/ (intense) sports  Listening to the body, avoiding overexertion, taking rest  Sports help in feeling fit but do not reduce IBD symptoms  **Movement barriers and adaptations**  Need to exercise more, time constraints, low motivation  Adjusting exercise to energy levels and IBD symptoms  Limited movement/ sports due IBD and/or fatigue  Limited movement/ sports due to comorbidity  Seeking advice on exercise  Financial barriers (*e.g.* quitting the gym due to cost)  **Supervised training and rehabilitation**  Sports with physiotherapist or personal trainer  Revalidation after surgery or for pain management  Exercise programs for COPD, IBD Fit program, lifestyle coaching | **250**  169  26  15  15  15  5  5  **74**  35  24  9  6  **117**  54  26  15  12  4  3  3  **326**  140  41  40  31  22  17  13  7  6  4  3  2  **57**  24  19  8  4  2  **120**  47  31  24  13  3  2  **28**  17  4  7 |
| Total respondents | 145 |  | 544 |
| **Poor sleep** | | | |
| **Information and professional guidance**  Information and advice (online)  Advice and support from a HCP  Advice  Personalized advice  More attention to poor sleep from HCPs  Information and advice on sleep and IBD  **Medical and therapeutic support**  Sleep medication  Help with mental health issues  New mattress  EMDR therapy for anxiety about poor sleep  Using weed  **Stress and mental well-being**  Reducing stress  Mindfulness, meditation, tools to relax  Talking about poor sleep  Poor sleep due to worrying  **Lifestyle and behavioral adjustments**  Getting more rest  Going to bed earlier  Exercise  Discipline  **Tools and support**  App  Peer support  **Sleep disruptions and external factors**  Poor sleep, poor falling asleep, not sleeping through the night  Poor sleep due to menopausal symptoms  Children sleeping through the night  Poor sleep due to night shifts, switching jobs to avoid night shifts  Poor day-night rhythm (due to work)  Poor sleep due to sleep apnea / overweight  Poor sleep due to disturbances  Poor sleep due to comorbidity  Poor sleep due to a snoring partner  Poor sleep due to a life event  **IBD-related sleep disruptions**  Poor sleep due to pain (IBD-related)  Poor sleep due to nighttime defecation  Poor sleep due to nighttime bathroom visits for emptying stoma bag  Poor sleep due to IBD symptoms  Poor sleep due to medication (side effects), stopping prednisone  **Fatigue and sleep quality**  Life dominated by fatigue, help with fatigue  Finding the cause of poor sleep  Feeling well-rested  Being tired enough to sleep well  **Other**  Tried a lot already  Do not know | **80**  33  31  10  4  1  1  **13**  7  2  2  1  1  **25**  12  9  2  2  **10**  5  3  1  1  **15**  13  2  **37**  10  6  6  4  3  3  2  1  1  1  **27**  9  5  5  4  4  **5**  2  1  1  1  **46**  4  42 | **Establishing a healthy sleep routine**  Going to bed on time, getting enough sleep hours  Regular sleep pattern  Less/ no screen time in evening  Regularity  **Relaxation and sleep preparation**  Yoga, mindfulness, meditation, relaxation exercises  Relaxing (in the evening)  Acupressure mat  **Lifestyle and dietary adjustments**  Considering food and drink intake (*e.g.* no caffeine, alcohol)  in the evening  Exercise  Healthier eating, losing weight to overcome sleep apnea  **Medical and alternative support**  Sleep medication  CBD oil, magnesium, herbs, vitamin supplements  Medication for pain, sleep disorder, menopausal symptoms  CPAP for sleep apnea, anti-snoring device  New bed, mattress, pillow, earplugs  **Addressing underlying causes of poor sleep**  Avoiding/ reducing (work-related) stress  Broken nights due to children / restless sleeping partner  Poor sleep due to stress  Poor sleep due to comorbidity, menopause, sleep disorder  **Poor sleep and IBD**  Poor sleep due to IBD symptoms, emptying stoma, frequent  nighttime bowel movements  Increased fatigue and need for sleep due to IBD  More IBD symptoms due to fatigue  **Professional help**  Sleep coach/ therapy, coach, online course  Psychologist, psychiatrist  Identifying causes and seeking help, participation in research  on poor sleep  **Self-management and coping strategies**  Resting during the day  Listening to the body  Stopping/ working night shifts  Adjusting daily rhythm, making choices in activities  Sleeping well  Accepting poor sleep  Monitoring sleep  Talking about poor sleep | **195**  86  72  20  17  **85**  47  35  3  **50**  36  9  5  **70**  45  12  5  5  3  **26**  11  8  4  3  **15**  8  5  2  **13**  10  2  1  **57**  28  6  5  5  4  4  4  1 |
| Total respondents | 195 |  | 339 |
| **Stress** | | | |
| **Information and professional guidance**  Advice and support from a HCP  Information and advice (online)  (Medical) psychologist/ mental health services  Guidance/ course (online) on coping with stress  Personalized advice  Information from patient organization  Personalized advice  **Stress management and coping strategies**  Reducing/ avoiding stress  Meditation / yoga / mindfulness / hypnotherapy  Learning to relax, more relaxation  Living more consciously, rest and routine  Preventing stress by setting priorities  **Tools**  App  **Work-related stress**  (Coping with) work-related stress  Cooperation of employer and understanding among colleagues  A different job with less stress  Help with work-life balance  **Social, emotional and practical support**  Help from/talking with loved ones  Peer support  Listening ear  Help with household tasks  **Stress triggers and external causes**  Stress caused by external factors, worries about others  More IBD symptoms due to stress  Stress in private life  Stress due to comorbidity  Accepting IBD  Stress outside the house due to toilet availability  **Persistent stress and challenges**  Stress remains, due to certain situations or personality  Stress remains, difficult to change  Stress remains, it is part of life  Insight into stress (situations)  **Other**  Nothing needed  Tried a lot already  Wants to stop antidepressants  Do not know | **95**  44  27  10  10  2  1  1  **31**  10  9  8  2  2  **11**  11  **23**  11  6  4  2  **7**  2  2  2  1  **22**  10  7  2  1  1  1  **38**  18  11  6  3  **25**  2  1  1  21 | **Avoiding and/or reducing stress**  **Stress reduction techniques and relaxation**  Yoga, mindfulness, meditation, relaxation/ breathing exercises, reiki  Exercise, sports  Relaxing, free time, hobbies, seeking distraction  Alternative, *e.g.* homeopathy, magnesium, cold showers,  valerian, acupuncture  Keeping a diary  Spirituality  **Mental and emotional support**  Psychologist, psychiatrist (antidepressants), coach  Therapy, *e.g.* EMDR, cognitive-behavioral therapy  General practitioner, nurse practitioner, social work  Talking about stress (with loved ones), seeking help  **Work adjustments**  Working less  Stopped working (retired, medically unfit)  Reducing work stress  Changing jobs  Addressing (work) stress at work  Work-life balance  **Mindset, lifestyle and perspective shifts**  Setting boundaries, saying no  Planning  Worrying less (about things you cannot control)  Routine  Putting things into perspective  Letting go  Making choices, setting priorities  Adjusting expectations  Living differently, changing lifestyle  Positive mindset  Living day by day  Accepting (IBD)  **Self-care and well-being**  Taking/ planning rest  Balance in life, conscious living  Healthy living, *e.g.* healthy diet, sleeping sufficiently  Taking good care of yourself, choosing for yourself, taking  control  Listening to your body  **Coping with the impact of stress on health**  Recognizing stress  More IBD-symptoms due to stress  Treatment for comorbidity  Performing activities gradually, staying calm  **Social and environmental factors**  Addressing the causes of stress, *e.g.* new home, relationships  Maintaining social contact  **Challenges in managing stress**  Difficult to change stress, character | **104**  **271**  112  80  68  9  1  1  **140**  66  30  8  36  **118**  33  29  24  22  10  9  **141**  34  23  22  12  12  10  9  5  4  4  3  3  **102**  66  14  11  6  5  **25**  9  8  4  4  **7**  5  2  **5**  5 |
| Total respondents | 164 |  | 537 |
| **Anxiety and depression** | | | |
| **Professional guidance and psychological support**  Advice and support from a HCP  Psychological support, (medical) psychologist, support from mental  health services  Attention for mental status and impact of IBD by HCPs  Advice and support from a HCP on coping with anxiety/ depression due  to flares  Advice on preventing anxiety and depression  EMDR therapy for reducing anxiety about IBD-related examinations/  care  EMDR therapy for anxiety and poor sleep due to IBD  **Information and advice**  Information and advice (online)  Information (online) about anxiety/ depression in relation to IBD  Information and advice about the long-term effects of IBD  Information about IBD and the desire to have children  Information and advice on anxiety/ depression as a side effect of  medication  **Tools**  App  **Social and peer support**  Social support (from relatives)  Peer support, IBD patient as a buddy  Daring to ask for help with anxiety  **Coping strategies and self-care**  Mindfulness, relaxation techniques  Improving physical condition, exercise  Taking good care of yourself, reflecting on feelings  Better coping with negative feelings and thoughts  Positive mindset  **Disease-related and external factors or IBD as triggers**  Help with anxiety and depression as causes of stress  Anxiety about IBD and the future and living with the disease  Anxiety due to menopause  Feeling down due to fatigue  **Barriers and limitations**  Difficult to change, part of personality and/or disease  Different living environment  Tried a lot already  **Other**  Do not know | **61**  41  11  5  1  1  1  1  **26**  16  6  2  1  1  **4**  4  **10**  6  3  1  **8**  2  2  2  1  1  **5**  2  1  1  1  **4**  2  1  1  **14**  14 | **Professional support and therapy**  Psychologist, psychiatrist, psychotherapist  Therapy, EMDR therapy, cognitive behavioral therapy,  haptonomy  Mental healthcare support  General practitioner and practice nurse  Coaching  Medical social work  **Medication and supplements**  Anti-depressants  Medication  Vitamin D, CBD-oil  **Relaxation and stress management**  Mindfulness, yoga  Relaxing  Meditation  Avoiding stress, anxiety, exhausting situations  Alternative therapy, *e.g.* horse therapy, hypnosis,  acupuncture, cold showers, reiki, biodynamic therapy  Breathing/ relaxation exercises  **Lifestyle and physical factors**  Sports, exercise  Taking rest  Good self-care, exercising, healthy eating, good sleep, going  outside  Reducing workload, changing jobs  Structure and planning  **Social and emotional support**  Talking to the environment, social support  Seeking distraction  Writing down feelings  **Mindset and coping strategies**  Accepting that life goes as it goes, pushing through, worrying  less, reducing negative feelings, trusting body, staying calm  Positive mindset  Relativizing  Discipline, keeping control, keeping situations small  (manageable)  Pushing boundaries, doing things that cause anxiety anyway  **Link between mental and physical health**  Anxiety/ depression causes stress  Feeling and understanding anxiety/ stress  Physical health, *e.g.* menopause, has a major impact on  mental health  **Mental health and IBD**  More IBD-symptoms when mental health declines  Medication causing depressive/ anxious feelings  Anxiety and stress about not having a toilet nearby | **125**  70  36  7  7  3  2  **33**  16  15  2  **61**  18  15  9  8  6  5  **35**  13  8  6  4  4  **23**  19  3  1  **25**  9  8  3  3  2  **6**  2  2  2  **5**  2  2  1 |
| Total respondents | 102 |  | 216 |
| **Lack of social support** | | | |
| **Increasing awareness and understanding**  Awareness and understanding among relatives/ others  Information and advice about living with IBD to share with relatives/  others  Awareness and understanding among relatives/ others, including fatigue  Writing a blog about living with IBD  **Information and professional support**  Information and advice (online)  Advice from a HCP  Being able to ask a HCP questions as a loved one  **Social and peer support**  Daring/ wanting to ask for help from others  Experiencing social support  Peer support  More support from the social environment  Experiences and stories from other IBD patients  **Tools**  App with a diary to track symptoms and functioning, to discuss with HCP  App  Tools to manage IBD together with loved ones, setting expectations  **Challenges in social support**  Difficult for informal caregiver to take IBD into account  Small social circle, building a new social circle  Living with IBD is lonely  Communicating better as a patient  Life changes  Not necessary for others to know how the person with IBD is doing  **Stress factors**  Reducing stress  Not always able to go to the toilet due to a busy home life, causing stress  **Other**  Nothing needed  Do not know | **39**  20  12  6  1  **14**  8  5  1  **9**  3  3  1  1  1  **3**  1  1  1  **12**  3  3  2  2  1  1  **2**  1  1  **7**  1  6 | **Seeking and receiving support**  Talk about IBD and share feelings  Experiences support from partner, family, parents, children,  friends and/or people who understand IBD  Support from HCP, *e.g.* psychologist, general practitioner,  coach  Peer support contact  **Challenges in social support**  Reducing/ avoiding stress (and people who do not  understand IBD)  Difficult for others to understand what living with IBD means  Experiences little social support/ lack of understanding (from  partner, parents)  **Proactive strategies for social support**  Asking for help  Setting boundaries  Asking for understanding  Maintaining social contacts  Discussing food choices  Following NLP practitioner training  **Importance of social support**  Social support in IBD/ during flare can make a difference | 45  19  14  1  **29**  1  2  11  **21**  11  4  2  2  1  1  **3**  3 |
| Total | 68 |  | 106 |

Abbreviations: CBD = Cannabidiol, CPAP = Continuous Positive Airway Pressure, EMDR = Eye Movement Desensitization and Reprocessing, FODMAP = Fermentable Oligosaccharides, Disaccharides, Monosaccharides and Polyols, HCP = Health Care Professional, HIIT = High-Intensity Interval Training

IBD = Inflammatory Bowel Disease, NLP = Neuro-Linguïstisch Programmeren
